# Ultrashort Echo Time for Improved Positive-Contrast Manganese-Enhanced MRI of Cancer

**DOI:** 10.1371/journal.pone.0058617

**Published:** 2013-03-04

**Authors:** Joris Tchouala Nofiele, Hai-Ling Margaret Cheng

**Affiliations:** 1 Department of Medical Biophysics, University of Toronto, Toronto, Canada; 2 The Research Institute and Diagnostic Imaging, The Hospital for Sick Children, Toronto, Canada; The Norwegian University of Science and Technology (NTNU), Norway

## Abstract

**Objective:**

Manganese (Mn) is a positive magnetic resonance imaging (MRI) contrast agent that has been used to obtain physiological, biochemical, and molecular biological information. There is great interest to broaden its applications, but a major challenge is to increase detection sensitivity. Another challenge is distinguishing regions of Mn-related signal enhancement from background tissue with inherently similar contrast. To overcome these limitations, this study investigates the use of ultrashort echo time (UTE) and subtraction UTE (SubUTE) imaging for more sensitive and specific determination of Mn accumulation.

**Materials and Methods:**

Simulations were performed to investigate the feasibility of UTE and SubUTE for Mn-enhanced MRI and to optimize imaging parameters. Phantoms containing aqueous Mn solutions were imaged on a MRI scanner to validate simulations predictions. Breast cancer cells that are very aggressive (MDA-MB-231 and a more aggressive variant LM2) and a less aggressive cell line (MCF7) were labeled with Mn and imaged on MRI. All imaging was performed on a 3 Tesla scanner and compared UTE and SubUTE against conventional *T*_1_-weighted spoiled gradient echo (SPGR) imaging.

**Results:**

Simulations and phantom imaging demonstrated that UTE and SubUTE provided sustained and linearly increasing positive contrast over a wide range of Mn concentrations, whereas conventional SPGR displayed signal plateau and eventual decrease. Higher flip angles are optimal for imaging higher Mn concentrations. Breast cancer cell imaging demonstrated that UTE and SubUTE provided high sensitivity, with SubUTE providing background suppression for improved specificity and eliminating the need for a pre-contrast baseline image. The SubUTE sequence allowed the best distinction of aggressive breast cancer cells.

**Conclusions:**

UTE and SubUTE allow more sensitive and specific positive-contrast detection of Mn enhancement. This imaging capability can potentially open many new doors for Mn-enhanced MRI in vascular, cellular, and molecular imaging.

## Introduction

Manganese (Mn), an essential metal for our body, is one of the earliest reported paramagnetic contrast agents for magnetic resonance imaging (MRI) due to its efficient positive contrast enhancement [1], [2]. Unlike gadolinium, a paramagnetic lanthanide ion approved for clinical use, manganese is an endogenous constituent and behaves like a calcium ion analogue that often acts as a regulatory cofactor in a number of important enzymes and receptors [3]. Its unique biological properties have lent themselves to various applications in functional and molecular imaging, most notably in imaging the liver [4], brain function [5], myocardial viability [6], and, more recently, cancer cells [7], [8]. In virtually all applications, the standard protocol is a *T*_1_-weighted pulse sequence to obtain positive signal contrast in areas of Mn accumulation.

One challenge to broadening the application of Mn-enhanced MRI is the need to increase detection sensitivity. Another challenge is to distinguish regions of positive contrast due to Mn accumulation from other tissues with inherently similar signal intensity. This is a dilemma in any kind of contrast-enhanced imaging, and the convention of using a pre-contrast image for comparison is, in many cases, impractical, especially when contrast accumulation occurs slowly and image misregistration becomes an issue. A method that does not require pre-contrast imaging and permits specific yet sensitive determination of contrast accumulation is desirable.

Ultrashort echo time (UTE) pulse sequences [9] have been applied to negative contrast iron oxide nanoparticles for this precise purpose: to improve detection sensitivity and specificity [10], [11]. In contrast to conventional “long” echo time sequences, UTE acquires signal very soon after excitation. This is particularly relevant to iron oxides, as it minimizes *T*_2_- and *T*_2_*-related signal decay and reaps *T*_1_-related signal enhancement, thereby turning the conventional “dark” contrast iron oxide into a “bright” contrast agent. Another unique feature of UTE is the capability to combine *T*_1_ and *T*_2_* effects synergistically by subtracting later echoes from the UTE image, thereby forming a subtraction UTE (SubUTE) image. In doing so, the *T*_2_*-related signal decay at the later echo is effectively reversed and added to the *T*_1_-related signal increase on the UTE image. This subtraction method not only enhances sensitivity but also provides background suppression, since only areas of contrast agent accumulation would experience large *T*_1_ and *T*_2_* effects. The handful of investigations into UTE has demonstrated its utility for more specific and sensitive imaging [10], [12], but these have focused primarily on negative-contrast iron oxides. The utility of UTE for imaging other MRI contrast agents remains largely unexplored.

In this study, our aim was to investigate the application of UTE and SubUTE to achieve more specific and sensitive positive-contrast detection of Mn enhancement. Although Mn is a *T*_1_-enhancing agent and does not suffer from the same issues as negative-contrast iron oxides, it stands to benefit from more specific and sensitive detection. Amongst paramagnetic contrast agents, Mn may be uniquely suited to benefit from the synergistic *T*_1_ and *T*_2_* effects of SubUTE imaging, owing to a relatively large effect on the transverse relaxation rate. This study investigates the feasibility and optimization of UTE and SubUTE detection of Mn through theoretical and phantom studies. A proof-of-concept study is demonstrated for Mn-enhanced cancer imaging, showing that UTE imaging provides sensitive detection of aggressive breast cancers, with SubUTE providing the best specificity.

## Materials and Methods

### Theoretical Studies

The UTE sequence is a spoiled gradient echo (SPGR) acquisition where signal intensity is described by the following steady-state equation:
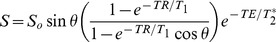
(1)where repetition time (TR), echo time (TE), and flip angle (θ) are adjustable imaging parameters; S_o_ is a global sensitivity factor; and *T*_1_ and *T*_2_* are longitudinal and effective transverse magnetization relaxation times specific to the tissue. The UTE signal can be modeled by setting TE to zero, thereby creating a purely *T*_1_-weighted image with no *T*_2_*-related signal decay. If an image acquired at a later echo time is subtracted from the UTE image, i.e. SubUTE(TE) = S(UTE) – S(TE), the resulting difference image provides synergistic *T*_1_ and *T*_2_* contrast.

In the presence of a MRI contrast agent such as Mn, *T*_1_ and *T*_2_* are approximated by:
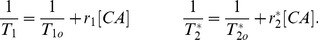
(2)where the subscript ‘o’ denotes baseline (i.e. no contrast agent), *r*_1_ and *r*_2_* are contrast agent relaxivities, and [CA] is contrast agent concentration. Contrast, or the signal difference induced by these *T*_1_ and *T*_2_* changes, can be determined from Eqs. [1] and [2] as follows:



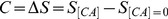
(3)Contrast was evaluated for UTE imaging using a very short TE, using a longer TE typical in conventional SPGR imaging, and using SubUTE. Optimal settings for TR, TE, and θ were investigated for various tissues with different baseline *T*_1o_ and *T*_2o_* relaxation times. This was accomplished by varying TR (6–100 ms), UTE (8–150 µs), long TE (0.01–100 ms), *T*_1o_ (300–3000 ms), *T*_2o_* (25–100 ms) to assess a range of Mn concentrations (0.001–10 mM).

### Phantom Studies

Manganese chloride (MnCl_2_) solutions were prepared by dissolving manganese (II) chloride tetrahydrate (Sigma-Aldrich Canada Inc., Oakville, ON, Canada) in water at various concentrations. The solutions were placed in borosilicate glass tubes with a diameter of 6 mm and height of 50 mm. The phantoms were imaged on a 3 Tesla MRI scanner (Achieva 3.0T TX, Philips Medical Systems, Best, The Netherlands) using a 32-channel receive-only head coil. The *r*_1_ and *r*_2_ relaxivities of MnCl_2_ were determined by measuring *T*_1_ and *T*_2_ relaxation times at different MnCl_2_ concentrations and calculating the regression slope. *T*_1_ was measured using a 2D inversion-recovery turbo spin-echo (TSE) sequence: inversion times (TI) = [50, 100, 250, 500, 750, 1000, 1250, 1500, 2000, 2500] ms, TR = 3000 ms, TE = 18.5 ms, TSE factor = 4, 60 mm field-of-view (FOV), 3 mm slice thickness, and 0.5×0.5 mm in-plane resolution. *T*_2_ were measured using a multi-echo spin-echo sequence: TR = 2000 ms, 32 echoes with TE = [7.63, 15.3, …, 244] ms, 60 mm FOV, 3 mm slice thickness, and 0.5×0.5 mm in-plane resolution. The relaxivity *r*_2_ was substituted for *r*_2_* in Eq. [2] as a first approximation for the phantom study. It is important to note that although this assumption is true for freely dispersed particles [13], [14], we may expect *r*_2_* to be higher than *r*_2_ when Mn is clustered inside cells, based on evidence from the iron oxide literature that *r*_2_*>>*r*_2_ upon cell internalization [15]–[17]. This point is discussed more fully in the Discussion.

The UTE sequence was run on the phantoms using a 3D steady-state gradient-echo sequence by varying TE (from 90 µs to 10 ms) and θ (10, 30, 50, and 70°) with TR fixed at 30 ms. As a preparation step, gradient channels were carefully calibrated to minimize off-resonance artefacts, and the tune delay of the coil was characterized for the shortest switching time. Multi-echo data with radial readout was then acquired with the following parameters: 60 mm cubic FOV, 3 mm slice thickness, and 0.5×0.5 mm in-plane resolution, and one signal average. For comparison, a conventional 3D SPGR acquisition was also performed, using the same TR and θ as in the UTE acquisition and setting TE = 2.83 ms (shortest).

### Breast Cancer Cell Studies

To investigate the value of UTE for positive-contrast imaging of Mn in biological systems, we labeled three different breast cancer cell lines with MnCl_2_. The three breast cancers were 231/LM2-4, MDA-MB-231, and MCF-7. The first two are more aggressive than MCF-7, with 231//LM2-4 being a highly metastatic variant of MDA-MB-231 generated in the Kerbel lab [18]. The other two cell lines were obtained from ATCC (American Tissue Culture Collection, Manassas, VA, USA). These cell lines will be hereafter referred to as LM2, MDA, and MCF7, respectively. All cells were grown in 1640-RPMI medium (Sigma-Aldrich Canada Inc., Oakville, ON, Canada) supplemented with 10% fetal bovine serum and 0.5% penicilin streptomicin. Cells were harvested by washing 80–90% confluent flasks with PBS and adding 0.05% trypsin EDTA (Gibco, Carlsbad, CA, USA). Cells were incubated for 1 hour with medium containing different concentrations of MnCl_2_ while they were in the exponential growth phase, after which they were rinsed with fresh medium and trypsinized as described above. Cell pellets were then prepared by centrifuge at 440 g for 10 minutes in the same borosilicate glass tubes used for phantom imaging. Immediately after, MRI was performed on breast cancer cell pellets on a 3 Tesla MRI scanner as described previously, using a range of TEs (90 µs to 10 ms) and θ (10, 30, 50, and 70°).

### Data Analysis

MRI data was transferred to an independent workstation for quantitative data analysis using in-house software developed in Matlab (v.7.8) (MathWorks, Natick, MA). To calculate the *r*_1_ and *r*_2_ relaxivities of MnCl_2_, regions of interest (ROIs) were outlined in the center of each glass vial on each image and a signal intensity curve obtained at every pixel location within the ROI as a function of TI (for *T*_1_ measurement) or TE (for *T*_2_ measurement). *T*_1_ relaxation time was quantified on a pixel-wise basis by fitting signal intensity to the function *A*×| 1–2× exp(−TI/*T*_1_)+exp(−TR/*T*_1_) |, where *A* and *T*_1_ are free parameters. *T*_2_ relaxation time was quantified on a pixel-wise basis by fitting signal intensity to a mono-exponential decay function added to a constant offset to account for noise. The mean *T*_1_ and *T*_2_ within the ROI were calculated along with the standard deviations. Relaxivities *r*_1_ and *r*_2_ were determined by linear regression analysis of the change in mean relaxation rates (1/*T*_1_ and 1/*T*_2_) versus Mn concentration. For all phantom and breast cancer cell imaging data, comparisons amongst different sequences were made on a ROI basis.

## Results

**Figure 1:**
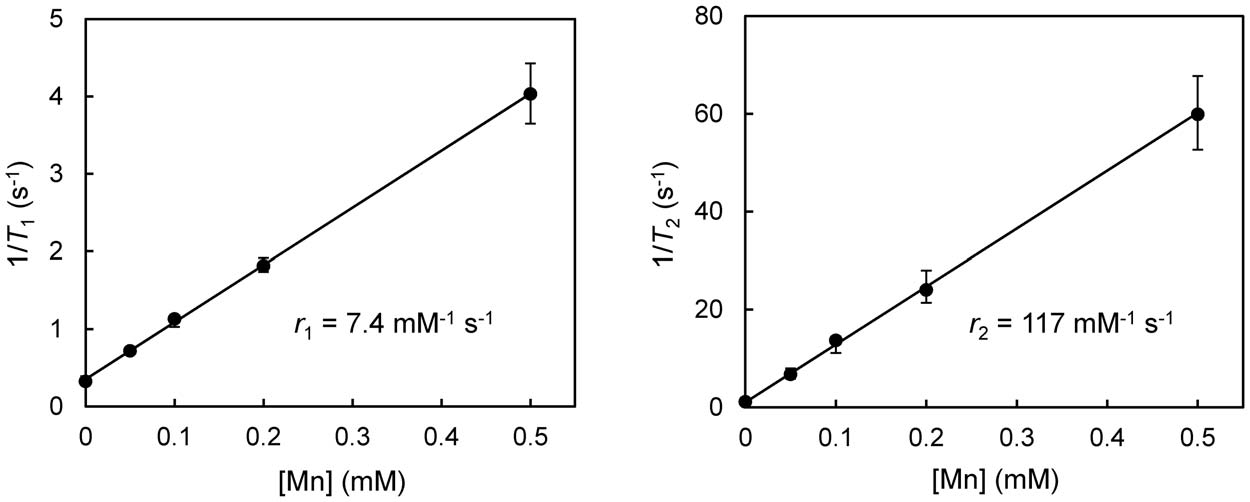
Relaxivity measurements of MnCl_2_ at 3 Tesla. Relaxation rates 1/*T*_1_ and 1/*T*_2_ versus MnCl_2_ concentration. Shown are mean values and standard deviations in each region-of-interest. Relaxivities *r*_1_ and *r*_2_ are calculated from linear regression slopes (*R* ^2^ = 0.9997 for *r*_1_; *R* ^2^ = 0.9995 for *r*_2_).

Measured relaxivity constants of aqueous MnCl_2_ at 3 Tesla are *r*_1_ = 7.4 mM^−1^s^−1^ and *r*_2_ = 117 mM^−1^s^−1^ (Figure 1). Note a much higher *r*_2_/*r*_1_ ratio relative to Gd-based paramagnetic agents where the *r*_2_/*r*_1_ ratio is roughly unity. The consequence of a large *r*_2_/*r*_1_ ratio is the presence of *T*_2_-related signal decay at higher contrast concentrations. While this is suboptimal for positive-contrast imaging and narrows the concentration range where enhancement can be reaped, it also provides an opportunity for additional contrast mechanisms offered by SubUTE imaging.

**Figure 2:**
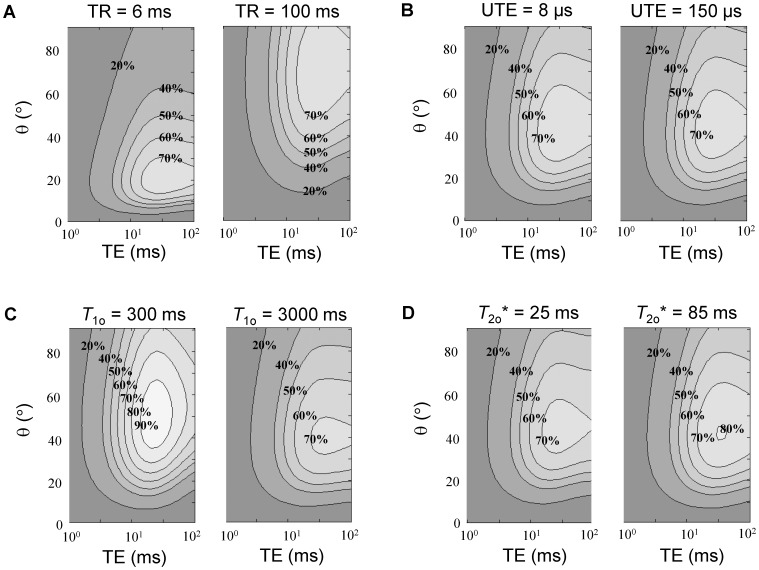
Relative contrast of SubUTE for different sequence parameters and baseline tissue properties. The relative contrast of SubUTE for different **A)** TR, **B)** UTE (i.e. shortest echo time), **C)** baseline tissue *T*_1o_, and **D)** baseline tissue *T*_2o_* for a Mn concentration of 1.0 mM. Where parameters are held constant, the following values were used in addition to measured relaxivities of MnCl_2_: TR = 30 ms, UTE = 90 µs, *T*_1o_ = 1000 ms, and *T*_2o_* = 47 ms. Relative contrast is expressed relative to the maximum contrast achieved on UTE. TR is seen to have the greatest influence on the optimal flip angle and TE (i.e. longer second echo).

**Figure 3:**
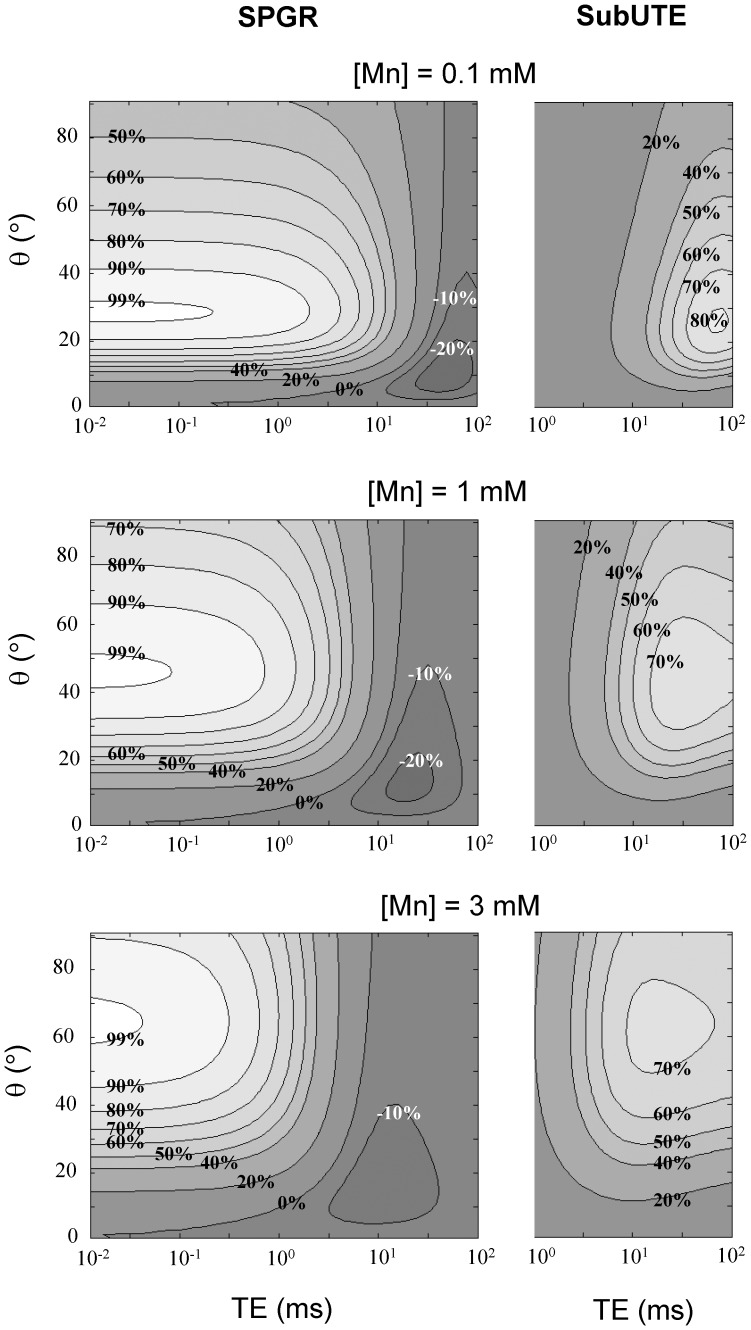
Relative contrast of conventional SPGR (including UTE) and SubUTE for different MnCl_2_ concentrations. Calculations were based on TR = 30 ms, measured relaxivities of MnCl_2_, and baseline tissue *T*_1o_ = 1000 ms and *T*_2o_* = 47 ms. To compare relative contrast of SPGR versus SubUTE, contrast isocontours are expressed relative to the maximum contrast achievable for each concentration. A √2 noise penalty was accounted for in the difference signal of a SubUTE image. For SubUTE plots, the first echo was set to 90 µs and the second longer echo is denoted as TE. It is seen that SPGR provides positive contrast at short TEs (including UTE) and negative contrast at longer TEs, whereas SubUTE provides strictly positive contrast.

**Figure 4:**
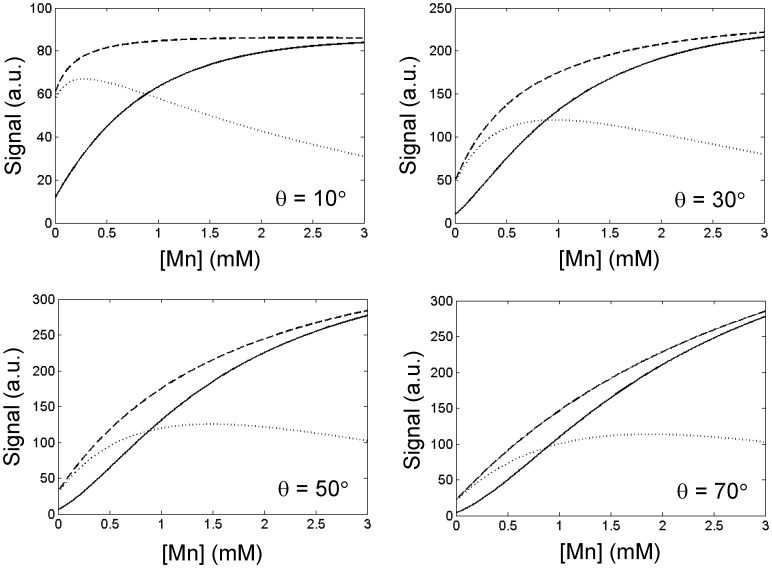
Theoretical signal intensity curves for UTE, SubUTE, and conventional SPGR. Signal intensity versus MnCl_2_ concentration for UTE (dashed line) with TE = 90 µs, SubUTE (solid line) with TEs = 90 µs and 10 ms, and conventional SPGR (dotted line) with TE = 2.83 ms. Calculations were based on TR = 30 ms and baseline tissue *T*_1o_ = 1000 ms and *T*_2o_* = 47 ms. A √2 noise penalty was accounted for in the difference signal of a SubUTE image. It is seen that higher flip angles θ provide greater positive contrast of high MnCl_2_ concentrations.

Simulations comparing the performance of conventional *T*_1_-weighted SPGR versus UTE (i.e. SPGR with a very short TE) and SubUTE are shown in Figures 2, 3, 4. Figure 2 illustrates SubUTE contrast as a function of TR, UTE, *T*_1o_ and *T*_2o_*. Note that only TR has a significant effect on the position of the peak positive contrast. Figure 3 compares the relative contrast of conventional *T*_1_-weighted SPGR versus SubUTE generated by Mn at concentrations of 0.1, 1, and 3 mM as a function of TE and θ. For illustrative purpose, a TR of 30 ms was chosen, as this value was consistent with the imaging requirements (i.e. imaging volume and slice thickness) of the in-vitro studies. In both Figures 2 and 3, contrast isocontours are expressed relative to the maximum contrast achievable for each concentration. Note also that although a range of TEs are shown up to 100 ms, the maximum relevant TE in any particular scenario cannot be larger than TR. The key purpose of Figure 3 is to show how to optimize contrast depending on the Mn range. In general, higher flip angles are necessary to maximize contrast at higher Mn concentrations, and, as shown in Figure 2, the optimal flip angle is tightly coupled to TR. The optimal TE for positive SubUTE contrast (i.e. the longer TE) and the optimal θ are relatively independent of the tissue baseline *T*_2o_* and exhibit a dependence on *T*_1o_ only at very low contrast concentrations less than 0.1 mM (data not shown). Figure 4 provides another perspective for comparing the different sequences by showing signal intensity as a function of Mn concentration. As seen also in Figure 3, a higher flip angle provides more linear signal enhancement at higher concentrations. Most importantly, Figure 4 clearly shows that both UTE and SubUTE provide much more linear and sustained signal enhancement, even in the regime where signal plateau cannot be avoided on conventional *T*_1_-weighted SPGR.

**Figure 5:**
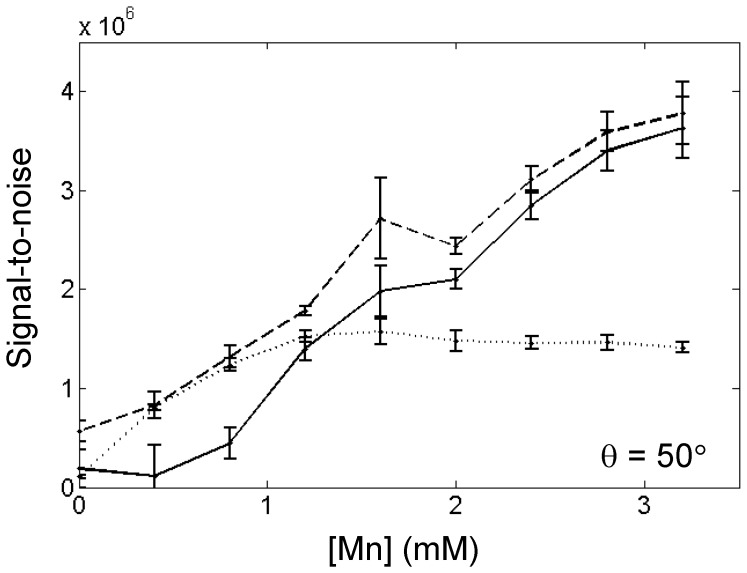
Phantom signal-to-noise curves for UTE, SubUTE, and conventional SPGR. Signal-to-noise in MnCl_2_ phantoms versus MnCl_2_ concentration for UTE (dashed line) with TE = 90 µs, SubUTE (solid line) with TEs = 90 µs and 10 ms, and conventional SPGR (dotted line) with TE = 2.83 ms. Shown are mean values and standard deviations in each region-of-interest.

Phantom results confirmed theoretical predictions. Conventional *T*_1_-weighted SPGR suffers from signal plateau and eventual signal decrease, whereas both UTE and SubUTE provide sustained and increasing positive contrast with higher Mn concentration. Figure 5 compares signal intensity on the different sequences for θ = 50°. The enhancement patterns are similar to simulation results.

**Figure 6:**
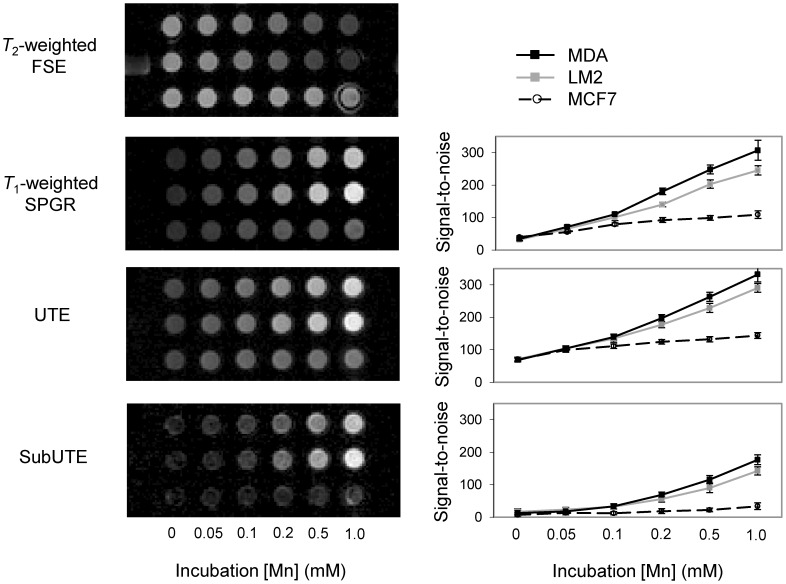
Detection of aggressive breast cancer cells on UTE, SubUTE, and conventional SPGR. Highly aggressive breast cancers LM2 (top row) and MDA (middle row) and less aggressive MCF7 (bottom row) incubated with MnCl_2_ at various concentrations are displayed as images (left column) and signal-to-noise (SNR) plots (right column). SNR plots present mean values and standard deviations in each region-of-interest. Cell uptake of Mn is seen as negative contrast on *T*_2_-weighted FSE and positive contrast on other sequences. UTE (TE = 90 µs) provides comparatively higher signal than conventional *T*_1_-weighted SPGR. SubUTE (TE = 90 µs and 10 ms) suppresses unlabeled or background tissue and also suppresses low [Mn] accumulation, and provides the best contrast between aggressive and less aggressive breast cancers.

Breast cancer cell imaging results are shown in Figure 6. Results are shown for θ = 50°, as this flip angle yielded the largest contrast changes across the different incubation concentrations. It is seen that the two aggressive cell lines, LM2 (top row) and MDA (middle row), take up much more Mn than MCF7 (bottom row) and, therefore, appear dark on *T*_2_-weighted FSE and bright on conventional *T*_1_-weighted SPGR. Note that because we limited the contrast dosage to a maximum of 1.0 mM Mn for labelling cells, we have not entered the signal plateau regime. Nonetheless, it is clear that even though conventional *T*_1_-weighted SPGR shows differences amongst the cancer cell lines, the UTE sequence provides slightly higher signal. By adding synergistic contrast mechanisms from *T*_2_* effects, the SubUTE sequence further emphasizes differences in Mn uptake between aggressive and less aggressive cancers. SubUTE provides the best distinction of aggressive breast cancers of all sequences, and only it provides simultaneous background tissue suppression.

## Discussion

The UTE sequence has been valuable for improving the visualization of normally dark-appearing iron oxide nanoparticles and tissue with short *T*_2_ and *T*_2_* (e.g. bone) by turning negative contrast into positive contrast. Although MnCl_2_ has *T*_1_ enhancing properties and does not share the same issues with negative contrast iron oxides on which UTE has been predominantly applied, the sensitivity and specificity of Mn-enhanced MRI can benefit from UTE imaging due to its characteristic high *r*_2_/*r*_1_ ratio. In this study, we investigated through theoretical, phantom, and breast cancer cell studies the detection of Mn on UTE and SubUTE imaging. It is shown that UTE and SubUTE significantly broaden the range of Mn concentrations over which positive contrast is sustained and remains linearly increasing, compared to conventional SPGR that suffers from *T*_2_* effects at higher concentrations. This capability not only improves detection sensitivity but can potentially provide a means for quantifying contrast concentrations. It is also shown that because SubUTE is a subtraction technique, background tissue is effectively suppressed, which may enable more specific determination of Mn accumulation in a contrast-enhanced study without the use of a pre-contrast baseline image. Furthermore, since Mn has a relatively high *r*_2_/*r*_1_ ratio, the SubUTE image can provide even greater contrast than UTE when *T*_1_ and *T*_2_* effects make comparable contributions, generally found at higher Mn concentrations; this is achieved by combining usually antagonistic *T*_1_ and *T*_2_* effects in a synergistic manner. Results in Mn-labeled breast cancer cells show that SubUTE achieves the best distinction of bright-appearing aggressive cancers from less aggressive cancers, which have similar contrast as background tissue.

Our theoretical study shows that optimal UTE and SubUTE contrast requires tuning the flip angle and TE to the range of Mn concentrations under consideration. For instance, higher concentrations of Mn translate to a lower *T*_1_, which means that a higher flip angle is necessary to achieve maximum positive contrast. The second echo used to form the SubUTE image should ideally lie in the 10 ms range. Both the optimal flip angle and TE are determined primarily by Mn concentration and TR. Note in Figure 3 that the lower contrast in the SubUTE signal relative to the SPGR (or UTE) signal is partly due to a √2 noise penalty in a SubUTE difference image and partly due to a strong *T*_1_ effect relative to *T*_2_*. Despite this lower sensitivity, SubUTE provides the greatest specificity of all sequences.

Phantom imaging confirmed theoretical predictions of signal plateau and eventual signal decrease on conventional SPGR, whereas UTE and SubUTE provided sustained positive contrast enhancement up to [Mn] = 3.2 mM, the maximum concentration tested. Optimal settings for flip angle and TE were similar to simulation results, with θ = 50° providing the optimum UTE and SubUTE contrasts over a wide range of concentrations.

Breast cancer cell imaging also confirmed that UTE offered better sensitivity and SubUTE better specificity of Mn-enhanced aggressive cells than conventional SPGR. There are, however, a few distinct differences from the phantom studies. First, the Mn concentration range of the incubation medium was much lower (up to 1 mM) for the cell-labeling studies, as our aim was to use as low a dose as possible on cells. Over this concentration range, theory predicts that conventional SPGR has not yet entered the signal plateau regime seen in the phantom study, which our cell imaging results confirmed to be the case. However, theory also predicts an optimal flip angle less than 50° for the lower concentrations used to label cells. This discrepancy suggests a higher *r*_2_* relaxivity than predicted, which is potentially caused by the accumulation of Mn to a greater concentration in cells and/or the formation of Mn clusters. To fully explain and accurately predict contrast mechanisms in a cellular environment, we need to understand how Mn distributes within these cells. A better understanding of these effects is important for future in-vivo applications but is outside the scope of this article. Despite this discrepancy, it is clear that even in cells, the SubUTE approach is able to reap synergistic *T*_1_ and *T*_2_* effects at higher Mn concentrations and provide the best detection specificity.

The discrepancy in the cellular environment noted above highlights a fundamental challenge when simulating the in-vitro or in-vivo environment, namely, the deviation of contrast agent distribution from the ideal of free dispersion. We used a measurement of *r*_2_ as a first approximation to *r*_2_*, mainly because accurate measurement of *r*_2_* is challenging and prone to variations from a variety of sources. However, this assumption will likely not hold once Mn is internalized. As reported in the iron oxide literature, *r*_2_*>>*r*_2_ when nanoparticles are compartmentalized in cells compared to free suspension [15]–[17]. Although Mn is not the same as iron oxides, we may postulate a similar phenomenon. That is, when Mn is internalized by cells, it does not distribute uniformly but, instead, accumulates in certain subcellular structures such as the mitochondria and forms clusters. As a result of compartmentalization, *r*_1_ will decrease due to limited water exchange and *r*_2_* will increase due to mesoscopic heterogeneities from bulk magnetic susceptibility effects. To optimize UTE and SubUTE imaging for cell studies, future work will need to investigate the distribution of Mn inside cells, how the distribution varies with different cell types, and the effect on *r*_2_ and *r*_2_* from varying Mn concentration and distribution.

Aside from the benefits of providing positive contrast and increased sensitivity under certain circumstances, perhaps the most practical advantage of SubUTE in vivo is background suppression, which effectively eliminates the need for a pre-contrast image. The convention of subtracting a pre-contrast baseline image from the contrast-enhanced image is cumbersome but is done to specifically locate areas of contrast agent accumulation. However, the baseline often cannot be perfectly co-registered, either because of patient movement or because contrast injection was done days earlier. With the SubUTE method, only one sequence is run and the contrast material can be localized wherever it has moved in the body and at any time, even days after contrast injection.

The use of Mn has gained renewed interest as an MRI contrast agent because of the potential to derived detailed physiological, biochemical, and molecular biological information [3]. In this study, we have presented a new application for Mn-enhanced MRI: assessing the aggressiveness of cancer cells. Our results demonstrate that our novel concept of using Mn to determine breast cancer aggressiveness is feasible and that the best distinction of aggressive versus less aggressive cells is achieved using SubUTE. Future studies will involve developing the proposed methodology in vivo, where safe contrast dosages and differences in the cellular environment (e.g. cell density) and deviation of relaxivities from the in-vitro scenario must be accounted for in optimizing UTE and SubUTE imaging. Beyond the demonstrated value of UTE for imaging cancer, it is our hope that with UTE as a new imaging capability for more sensitive and specific detection of Mn, many more applications associated with Mn-enhanced MRI will be explored and developed to characterize biological systems.

### Conclusions

In this study, we have introduced a new application of UTE and SubUTE imaging for Mn-enhanced MRI. Although Mn is inherently a positive-contrast *T*_1_ agent, UTE and SubUTE improve detection sensitivity over conventional SPGR, and they enable sustained and linear positive contrast enhancement over a wide range of Mn concentrations, even at high concentrations where signal would normally plateau or decrease. The SubUTE sequence provides additional specificity of Mn accumulation by eliminating background tissue and even increased sensitivity under certain circumstances by combining usually antagonistic *T*_1_ and *T*_2_* effects. Contrast localization on SubUTE does not require a pre-contrast baseline image, which is a significant advantage in any contrast-enhanced examination.
